# The fabrication of freestanding complex oxide membranes: Can we avoid using water?

**DOI:** 10.1557/s43578-024-01461-y

**Published:** 2024-10-24

**Authors:** Dae-Sung Park, Nini Pryds

**Affiliations:** Department of Energy Conversion and Storage, Technical University of Denmark (DTU), Building 310, 2800 Kgs. Lyngby, Denmark

**Keywords:** Freestanding oxide, Complex oxide, Thin films, Epitaxial lift-off, Release and transfer, Sacrificial layer

## Abstract

Recent advances in fabricating scalable two-dimensional or freestanding functional materials have shown promise for their use in modern silicon-based electronics and future technologies. A growing interest is in creating freestanding complex oxide membranes using new methods like epitaxial lift-off and mechanical exfoliation to enhance their quality and integrity. Despite these advances, it remains challenging to consistently produce high-quality freestanding oxide membranes on a large scale for practical use. This perspective paper provides an overview of release-and-transfer techniques for fabricating freestanding single-crystalline complex oxide layers, which are initially grown epitaxially. Specifically, we systematically explore the advantages and disadvantages of water-assisted exfoliation of freestanding oxide layers, which have been widely adopted using a water-soluble sacrificial layer in recent years. Furthermore, we compare this approach with other methods to navigate future directions in oxide layer transfer technology, considering material selections, fabrication processes, and functionalization strategies.

**Graphical abstract:**

**Figure:**
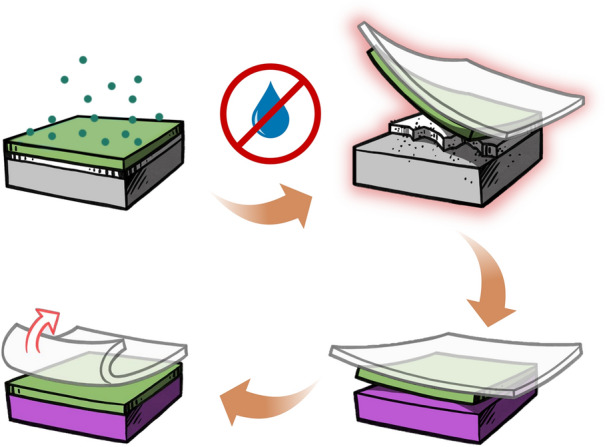
Seeking a water-free epitaxial lift-off process: (1) Growth: An epitaxial film is grown on a sacrificial layer placed on a single-crystal substrate (top left). (2) Release: The film is released from the substrate without using a water-based sacrificial layer (top right). (3) Transfer: The film is transferred onto a new platform (bottom right). (4) Support Removal: A polymer support is removed from the film (bottom left).

## Introduction

**Figure 1:**
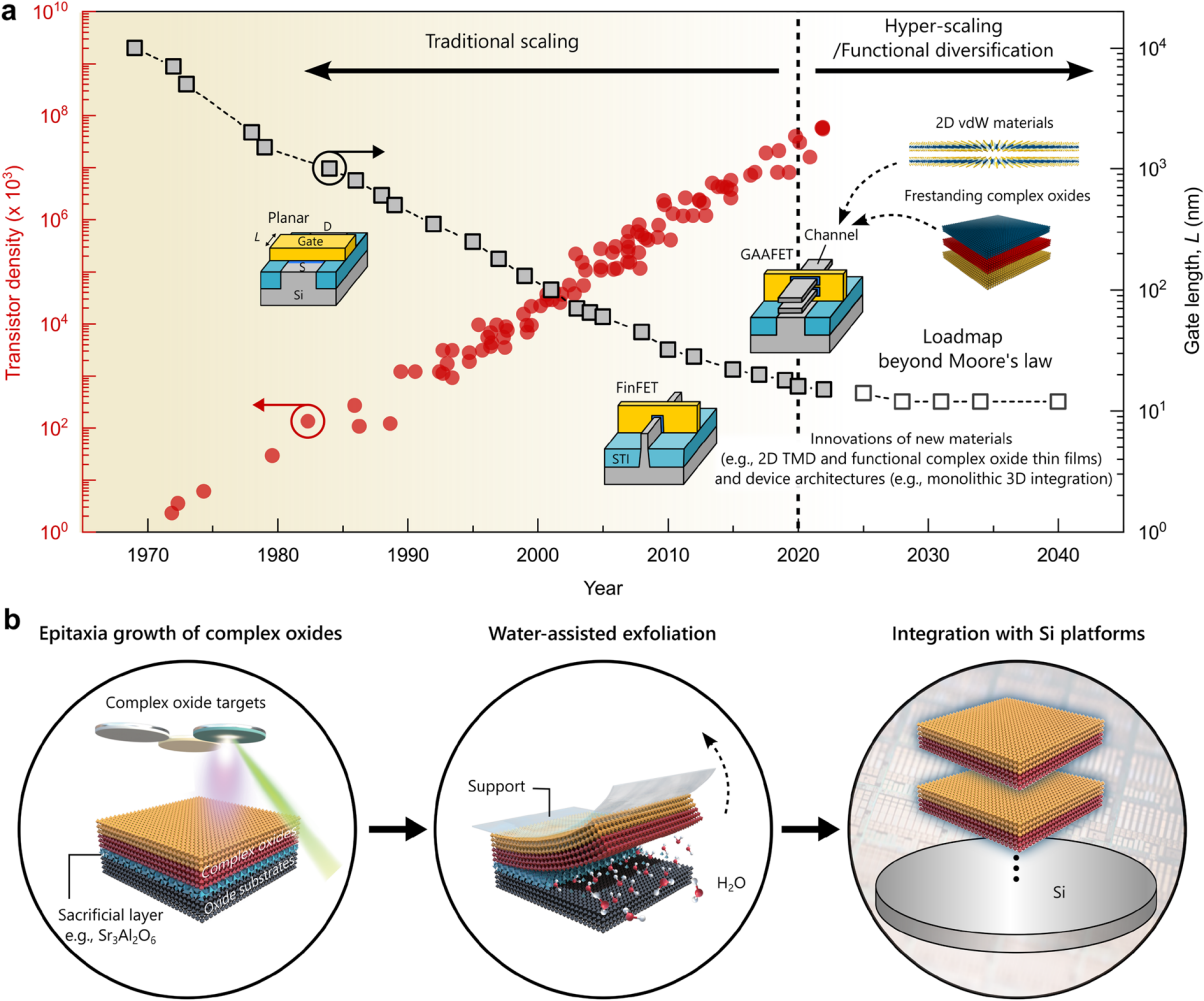
(a) The evolution trend of technology nodes for the CPU transistor density (left side) and MOSFET gate length (right side), combined with the IRDS roadmap beyond Moore’s law [15]. To increase the number of transistors per chip, CMOS scaling has been continuously undertaken over the last five decades through the innovations in the FET structures, evolving from 2 to 3D designs (e.g., FinFET and GAAFET). For ‘More Moore’ and functional diversification, 3D heterogeneous integration technology is promising, including the development of alternative channel materials (e.g., 2D transition metal dichalcogenide (TMD) and freestanding single-crystalline complex oxide layers) and new device architectures. (b) Water-assisted epitaxial lift method using water-soluble sacrificial layer [16]: (i) the growth of a single-crystalline epitaxial oxide heterostructure, consisting of functional complex oxide layer and water-soluble sacrificial layer, on an oxide single crystal substrate, (ii) the exfoliation of the grown complex oxide layer by dissolving the sacrificial layer in water, and (iii) the integration of released complex oxide layer onto Si platforms. In recent years, this method has been most widely used for fabricating freestanding complex oxide membranes.

Moore’s Law, established in 1965, forecasts that the number of components in an integrated circuit will double approximately every two years [1]. Following this principle, modern silicon (Si)-based complementary metal–oxide–semiconductor (CMOS) technology has significantly progressed by enhancing device integration density. This means that increasingly more transistors are being compacted into smaller chip areas by reducing their size. However, the size scaling of planar Si transistors reached its physical limits with smaller gate lengths, significantly causing adverse effects on device performance, such as high leakage current, short-channel effects, the thickness fluctuation of Si surface, and quantum tunneling [2–8]. In the 2000s, the dimensional scaling slowed with the conventional solid-state technology, aided by the strained Si, Si-Ge channels, high-*k* gate dielectrics, and non-planar fin field-effect transistors (FinFET) [9–12]. Later, the transistor density was further increased by developing high-aspect-ratio tall fins, known as gate-all-around FET (GAAFET), enabled by advancements in process integration and patterning techniques [13, 14]. However, further CMOS downscaling needs to surpass beyond geometrical and effective scaling. Nearly 60 years after the postulation of Moore’s law, this continuous evolution has reached the fundamental limit of dimensional scaling (e.g., up to 10 nm) in conventional Si-based MOS technology [Fig. 1(a)] [15]. Hence, for future technology innovation beyond the post-Moore era, achieving alternative channel nanomaterials and integration technologies is in strong demand as an essential engineering step along with the development of hyper device scaling and functional diversification, as shown in Fig. 1(a). This is particularly for realizing the next-generation electronic operations for complex integrated circuits and computing systems such as the Internet of Things (IOT), artificial intelligence, and high-performance computing.

Recent advances in fabricating freestanding single-crystalline oxide membranes [16] (e.g., complex oxide thin layers) are emerging with a range of functional properties with high tunability, e.g., exceptional electronic [17], dielectric [18], optoelectronic [19], mechanical [20–22], energy conversion [23, 24], and topological properties [25]. This is because these freestanding oxide membranes can be easily integrated with existing technological platforms (e.g., Si and flexible substances). When these freestanding layers are stacked or twisted, synergistic and/or exotic effects can arise at the heterojunctions/interfaces [25, 26], for example, creating multiferroic-like membranes by stacking ferroelectric and ferromagnetic layers together. Therefore, the heterogeneous integration of such functional freestanding membranes has been regarded as a promising strategy not only for enhancing device performance but also for facilitating the design of next-generation electronics and device integrations alongside current CMOS technologies [18].

Complex transition metal oxide thin films have garnered significant attention due to their versatile functionalities, e.g., piezoelectricity/ferroelectricity [27–29], high-temperature superconductivity [30], ionic conductivity [31, 32], colossal magnetoresistance [33], and room-temperature magnetism [34, 35]. These emergent properties arise from the complex interactions between the charge, spin, orbital, and lattice degrees of freedom, which are highly tunable through epitaxial strain. The heteroepitaxy of complex oxide thin films offers a film quality comparable to single crystals. A crucial factor in producing high-quality epitaxial films is the selection of the underlying substrates that appropriately match the lattice of upper film layers. The heteroepitaxial strain of approximately 1 – 3% is commonly observed in epitaxial oxide thin films. The correlated structural and electronic properties of complex oxide thin films are intimately linked to the interfacial constraints and interactions with the single crystal substrates. Such epitaxial strain can alter the physical properties of complex oxide thin films, as observed in phenomena like near room-temperature metallicity of epitaxially strained metal-insulating transition oxide thin films, e.g., VO_2_/TiO_2_(001) [36] and affect the ferroelectric polarization, e.g., BiFeO_3_/SrTiO_3_(001) [37]. Thus, identifying the optimal degree of lattice mismatch between the film and substrate is essential in fabricating tailored functional epitaxial oxide thin films.

These constraints linked to the substrates significantly limit the use of epitaxial oxide films in silicon-based technology and other widely used substrate platforms. To overcome these challenges, the use of advanced growth strategies, e.g., employing appropriate buffer/passivation layers in the case of Si to prevent SiO2 formation or to mitigate thermal stress, or alternative layer transfer techniques, has been suggested.

Alternatively, the epitaxial lift-off technique, which employs a sacrificial layer, has been developed to produce freestanding film layers of single-crystalline transition metal oxides [38]. These films can then be transferred to any substrate. Among the developed epitaxial lift-off techniques, a common approach to achieve these freestanding epitaxial oxide thin films is to utilize a sacrificial oxide layer inserted between the functional oxide film layer and a single crystal substrate. The crucial step of this approach is the selection of the sacrificial layer and selecting suitable etching solutions. The lattice parameter of the sacrificial layer must be similar to those of both the desired upper thin film layer and substrate to facilitate epitaxial growth without introducing significant defects (e.g., line and planar defects). It shall be noted that an ideal etching solution should only dissolve the sacrificial layer and should not have any effect on the grown upper film layers. One of the common sacrificial layers is cubic Sr_3_Al_2_O_6_ (SAO, space group *Pa* $\overline{3 }$, *a* = 15.844 Å) [38], which can grow epitaxial complex oxide thin films, e.g., SrTiO_3_ (STO), SrRuO_3_ (SRO), and La_1−*x*_Sr_*x*_MnO_3_ (LSMO) [38–41], and release them from single crystal substrate (e.g., STO). The most significant advantages of using SAO are (i) similar cation sublattice parameter [a* (*a*/4) =  ~ 3.96 Å] with that of prototypical ABO_3_ perovskite materials for epitaxial film growth and (ii) its water solubility, which subsequently allows for easy etching processes accessible in the laboratory. The heteroepitaxy of SAO-based heterostructure on oxide single crystal substrate, water-assisted lift-off, and integration of freestanding oxide layers are schematically shown in Fig. 1(b). Some of the challenges associated with SAO include (i) the large lattice constant of SAO does not align well with the growth of many functional complex oxides (e.g., LSMO), (ii) difficulty in fabricating high density (>75%) target materials for pulsed laser deposition (PLD), which can significantly causing the formation of particles during the deposition, and (iii) a slow etching rate of SAO in a solution, e.g., typically requiring more than 1–2 h to etch a 20-nm-thick SAO sacrificial layer inserted in most heterostructures.

One way to minimize the lattice mismatch is to adjust the lattice parameter of the water-soluble layer, for example, a decrease (an increase) in the lattice parameter of SAO by doping Ca (Ba) into Sr-sites [42]. A systematic investigation of tuning the crystal structure and the membrane-released features of the SAO-embedded heterostructures was recently carried out, comparing various thicknesses of the sacrificial layer, lift-off time, and different polymer supports. The results show that the epitaxial lift-off process for obtaining millimeter-sized freestanding membranes typically requires a lengthy release duration of several hours for etching 20-nm-thick inserted SAO layer in most heterostructures, often extending up to 30 h [42]. More recent advances have been made by introducing a new water-soluble sacrificial layer, “super-tetragonal” Sr_4_Al_2_O_7_ (SAO_T_, a* =  ~ 3.87 Å and c* =  ~ 4.32 Å) [43], which is a member of the SrO–Al_2_O_3_ family. This compound exhibits a significantly higher dissolution rate, approximately ten times faster (a few tens of minutes) than that of its counterpart, Sr_3_Al_2_O_6_. The high dissolution rate of SAO_T_ is most likely related to the discrete Al–O networks and higher concentration of water-soluble Sr–O species in this compound. It was suggested that SAO_T_ could be more adaptable for accommodating epitaxial strain with widely employed single crystal oxide substrates (e.g., STO and scandate-based perovskites) due to its low-symmetry crystal structure [43]. This characteristic may allow for adapting the sacrificial layer to different epitaxial layers with high tunability of the lattice constants. These advantages of crystalline coherence and a defect-free interface observed in perovskite ABO_3_/SAO_T_ heterostructures are suggested to be crucial for preventing the formation of microcracks after exfoliation [43]. Thus, the high strain compatibility and water solubility of SAO_T_ make it an ideal sacrificial layer for producing large-sized crack-free freestanding oxide membranes. However, the methods mentioned above for epitaxial lift-off of freestanding perovskite thin film layers primarily rely on water-based processes involving the dissolution of (Ca,Sr,Ba)_x_Al_y_O_z_ sacrificial thin film layers.

While the water-assisted exfoliation of freestanding oxide thin films represents an effective and easy-access approach, challenges persist with this method. Specifically, questions arise regarding the properties of freestanding complex oxide membranes when the film materials are sensitive to water exposure. In the following sections, we review these challenges and offer a perspective on potential future directions in the field. The primary objective of this perspective paper is to explore alternative solutions for the epitaxial exfoliation of freestanding oxide layers that avoid the use of water-assisted processes, thereby preserving the functional properties of these layers.

## Strategies for water-free process

### Remote epitaxy

**Figure 2:**
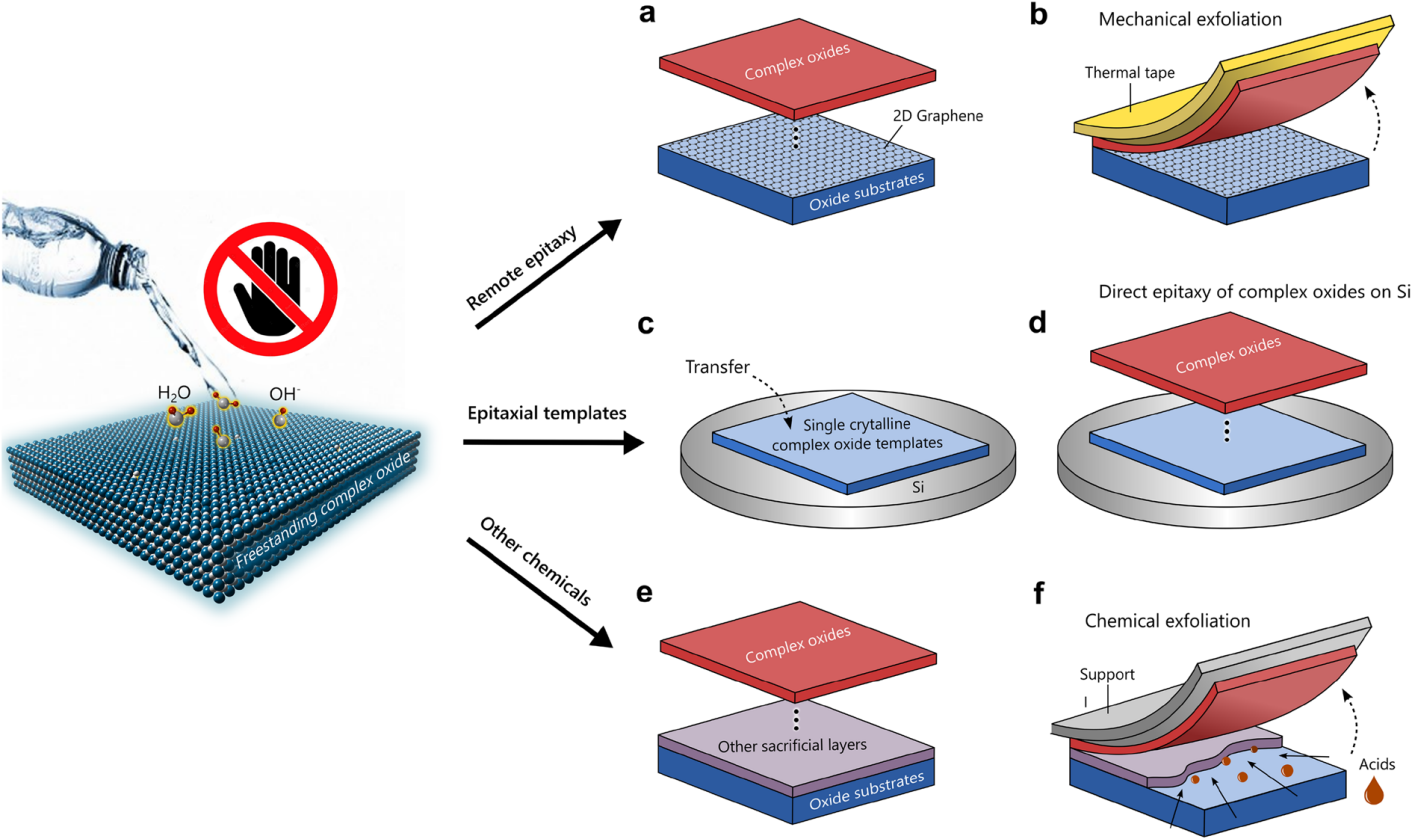
Possible water-free lift-off methods for fabricating freestanding single-crystalline complex oxides membranes and their integrations onto Si. (a, b) Remote epitaxy of single-crystalline complex oxides on oxide single crystal substrates covered by a 2D graphene sheet through electrostatic potential of the underlying layers/substrates (a) and the subsequent mechanical exfoliation of the oxide layers (b). (c, d) A direct epitaxial growth of complex oxides on Si platform where a single-crystalline complex oxide membrane is initially integrated as an effective epitaxial template. Prior to the epitaxial growth of a desired functional complex oxide layer, an appropriate epitaxial template on Si needs to be selected considering the crystal structure and lattice mismatch. (e, f) Heteroepitaxy and chemical exfoliation of complex oxide layers by employing other sacrificial layers which are soluble in or nearly water-free chemical solutions (e.g., HF acids). For this method, careful attention on the appropriate selection and concentration of etchants are required to avoid chemical damage and corrosion to desired upper functional complex oxide layers during the etching processes.

The concept of remote epitaxy is to epitaxially grow thin films on the surface of a single crystal substrate, covered by an atomically ultrathin 2D material such as graphene [Fig. 2(a)] [44]. During film growth, remote interaction between the deposited adatoms and the substrate occurs through the electrostatic potential of the substrate surface, which extends across the atomic scale 2D interlayer (<1 nm). This interaction aligns the adatoms along the crystal lattice of the underlying substrate. This technique enables the epitaxial growth of thin films and their subsequent mechanical exfoliation from the substrates [Fig. 2(a, b)], resulting in freestanding thin film layers. It is an evolution from the conventional epitaxy process, where materials are grown directly on a crystalline substrate to form an overlayer that aligns with the crystal lattice and orientation of the underlying substrate. The unique aspect of remote epitaxy is that it allows the epitaxial growth of crystalline films across a 2D-layer, which has no out-of-plane atomic bonds, resulting in upper epilayers with weak interlayer interactions. The interlayer, often made of graphene, is typically just one or two atoms thick (≤1 nm), which is thin enough for the upper epilayer to interact remotely with the crystal lattice of the underlying substrate during film growth while remaining non-intrusive enough to detach the grown epilayer by a handling layer (thermal release tape). Some of the materials including GaN, [45–47], AlN [48], GaAs [49, 50], and Ge [50] films, just to name a few, have been used for remote epitaxy.

The primary benefit of remote epitaxy is its capability to facilitate the direct fabrication of freestanding high-quality single-crystalline film layers as epilayers without requiring primary chemical treatments. These standalone layers can be moved to different surfaces for integration or used as separate functional membranes. This is especially useful in electronics and photonics, where the quality and flexibility of the materials are essential. Due to the chemical flexibility and diverse functions of perovskite complex oxides, applying the remote epitaxy to these materials is very promising. Various single-crystalline oxide films were remotely grown on graphene-coated single crystal substrates using conventional oxide molecular beam epitaxy (MBE) [49, 51], followed by mechanical exfoliation of several important oxide materials including perovskite STO, perovskite BaTiO_3_ (BTO), spinel CoFe_2_O_4_ (CFO), and garnet Y_3_Fe_5_O_12_ (YIG) [44]. Recently, a hybrid MBE approach has been developed, employing metal suboxides and an oxygen source [52]. This new growth method oxidizes the metals right at the growth front, allowing for a gentler oxygen source to avoid the modification/damage of the graphene layer. This innovative approach results in 46-nm-thick SrTiO_3_ epitaxial films that maintain self-regulating cation stoichiometry. The MBE-grown films were successfully exfoliated and transferred onto different substrates (e.g., Al_2_O_3_). However, using pulsed laser deposition (PLD), remote epitaxy shows significant challenges with the 2D graphene interlayer. For the PLD growth of most complex oxides (e.g., LSMO and SRO), employing high oxygen pressures is typically required, which can easily lead to the oxidation of the graphene layer. This is problematic as the formation of graphene oxide causes the grown film layers to be mechanically non-exfoliated from the 2D-layer/substrates. Additionally, the high kinetic energy of the particles in the plasma plume can cause severe damage to the graphene. Recently, it was shown that managing the growth kinetics of STO by reducing the laser spot size and using high argon pressures in the PLD chamber can solve these challenges [53]. This approach preserves the graphene interlayer with a low defect density during PLD while enabling the epitaxial layer-by-layer growth of oxide layers.

A key prerequisite for remote epitaxy is the fabrication of high-quality, defect-free, and uniform 2D layers on oxide single crystal substrates prior to film growth. For example, metallic or organic residues and adverse defects, e.g., wrinkles, tears, holes/voids, etc., unintentionally form during the transfer of the graphene layer onto oxide substrates. These foreign contaminants, defects, and pinholes significantly affect the subsequent film growth, leading to issues, e.g., the growth of a non-uniform film layer and/or polycrystalline film growth. Especially, when films are grown on the uncovered areas of the underlying substrates where microscale pinholes are initially formed, this causes difficulties in exfoliating the grown films from the substrates. This results in a spalling failure of membranes as forming flakes. This ultimately limits reproducibility and reliability for the large-area and uniform exfoliation of grown films. Kum et al. [44] found that using multiple layers of graphene, such as a bilayer, enhances remote epitaxy and the following exfoliation process by (i) protecting the oxygen-free graphene from the oxide substrates underneath during film growth and (ii) increasing the area yield of exfoliation. This approach can be applied to virtually any material system as long as the graphene layer does not completely screen the electrostatic potential of the underlying material, e.g., atomic potential fields of approximately 10–60 meV occur across a graphene monolayer or bilayer between the upper STO and the underlying TiO_2_-terminated STO [44].

Remote epitaxy enables the transfer of freestanding oxide membranes without using water. This method is advantageous as water can degrade or largely modify the intrinsic functional properties of engineered films and substrates sensitive to moisture, like phase transformation, oxygen vacancies, and surface charge polarization [54–59]. So far, this approach has been conducted only with graphene interlayers. Thus, it could be extendable with other transferrable 2D van der Waals (vdW) materials, which sustain high-temperature film growth, e.g., hexagonal boron nitride and MXenes [60, 61]. Crucially, for the successful remote epitaxy and subsequent exfoliation of epitaxial oxide thin films, we need to address and resolve certain issues, such as the oxidation and damage of 2D interlayers. This involves developing specific growth procedures. For instance, in PLD, this could mean adopting a two-step film growth process that starts with creating an initial oxygen-free buffer layer under reduced oxygen conditions, followed by the growth of the main functional oxide film under suitable oxygen partial pressure.

### Using freestanding epitaxial templates on Si

Integrating and processing single-crystalline oxide films into silicon-based electronic device platforms is crucial for practical applications of functional oxides. For example, several attempts have been made to grow single-crystalline epitaxial ABO_3_ perovskite STO (and similar perovskites) thin films directly on Si. Using conventional methods to grow oxide films on silicon substrates often leads to the formation of polycrystalline or amorphous STO, which poses a significant challenge. The major issue arises from the “conflict” of oxygen between the oxide film and Si substrate, which happened during the growth at high deposition temperatures of 600–800 °C. The insufficient oxygen content during film growth prevents the formation of high-quality, stoichiometric STO films on silicon substrates. Thus, over the last decades, the control of oxide/Si interface remains one of the significant challenges for oxide epitaxy on Si with sophisticated synthesis processes [62]. Additionally, compared to the cost of silicon-based substrates (approximately up to $30 for a 4-inch Si wafer in 2024) [63], complex oxide substrates like STO are significantly more expensive—in the range of few times—a few tens times higher, depending on suppliers. This higher cost is due to the excellent lattice quality of the single crystal maintained throughout their entire volume, ranging from 0.1 to 5 mm thick. This significant cost difference highlights the need to use more affordable oxide materials while ensuring that high-quality crystallinity and functionality are maintained in the critical thin top layer of silicon-based heterostructures tailored explicitly for specific applications. To overcome these issues, researchers are actively seeking possible solutions for growing STO films epitaxially on Si, which is an active area of research [64]. For example, by utilizing the epi-STO/Si platform, it is possible to achieve epitaxial integration of various complex oxide systems on Si-based semiconductors [65, 66]. Therefore, a crucial technological step involves effectively growing an epitaxial ABO_3_ perovskite STO template layer on silicon using methods such as MBE or PLD. The need for an STO template arises because the cubic lattice of STO (*a* = 3.905 Å) matches well with a wide range of functional complex oxides, such as high-k dielectrics, piezoelectrics, and ferromagnets. This compatibility facilitates their integration into various applications. To minimize the initial interface reaction during STO epitaxy on silicon, complex and precise growth procedures are necessary. One such method is a two-step growth process [66]: (i) initially growing a buffer layer at a low partial oxygen pressure (P_O_) of approximately 10^−8^ mbar and a low temperature of 350 °C, followed by (ii) the primary film growth at a higher P_O_ of approximately 10^−6^ mbar and a relatively high temperature of 550 °C.

Alternatively, it has been shown very recently that graphene can act as a diffusion barrier layer between the STO thin film and the Si substrate, making the integration of functional transition metal oxides into Si-based technology feasible [67]. Another recent work shows a method to grow high-quality single-crystalline thin films on Si substrates that are initially integrated with SrTiO_3_ or other single-crystalline oxide membranes [Fig. 2(c)] [54, 68]. Freestanding SrTiO_3_ membranes were successfully used as an epitaxial templet for the subsequent epitaxial growth of various functional thin films such as SrNbO_3_, SrVO_3_, TiO_2_, and dichalcogenide 2D superconducting FeSe2 on Si or other semiconductors as schematically shown in Fig. 2(d). This study demonstrates a new pathway toward realizing multifunctional single-crystalline oxide heterostructures incorporated with Si-based semiconductor device architectures. In addition, this method offers benefits for preserving the water-sensitive functional properties of complex oxide thin films. However, it requires a careful film growth optimization process, e.g., considering thermal mismatch and chemical diffusion.

### Other chemical lift-off methods

Chemical etching is an alternative method to reduce water reactions and minimize adsorption on freestanding complex oxide membranes. This technique involves applying sacrificial layers that dissolve exclusively in specific chemical solutions [Fig. 2(e, f)]. This method has become popular over the past few decades for undercutting and selectively releasing high-quality epitaxial films. It is primarily used to design metal–oxide–semiconductor and micro-electro-mechanical systems (MEMS) device architectures. It has been possible to achieve large-area conventional semiconductor film layers, e.g., Ga_1−*x*_Al_*x*_As/GaAs, from the growth substrates by dissolving sacrificial layers with appropriate etchants, e.g., SiO2 and AlAs in dilute hydrofluoric (HF) acid [69–71]. Intensive research has been conducted using various complex oxides as sacrificial layers to create freestanding membranes from epitaxial platforms. For example, epitaxial SrRuO_3_ (SRO) thin films were coherently grown on STO (001) single crystal substrate. After film growth, the STO substrate was selectively etched with an acid (e.g., 50% HF: 70% HNO_3_:H_2_O = 1:1:1) to make a lift-off of upper SRO thin film which is insoluble in acids [72]. On the other hand, SRO can be dissolved by using aqueous solutions (e.g., 0.4 mol/l H_5_IO_6_ for 20 nm/sec, 0.4 mol/l NaIO_4_ for 2 nm/sec, and 0.4 mol/l NaClO_3_ for slow etching – several hours) [73, 74]. These solutions change the oxidation state of RuO_2_ (Ru^4+^) to volatile RuO_4_ (Ru^8+^) and dissolve the remaining SrO_2_: SrRuO_3_ (s) + 2H_3_O^+^ + 2I ${\text{O}}_{4}^{-}$→Sr_2_ (aq) + RuO_4_ (aq) + 3H_2_O + 2I ${\text{O}}_{3}^{-}$. Brownmillerite SrCoO_2.5_ (SCO, pseudocubic *a*_pc_ = 3.905 Å and *c*_pc_ = 3.9363 Å) has been presented to be a promising sacrificial layer. It can be dissolved in acetic acid, vinegar, or carbonated drinks for relatively short etching times, 3 – 10 min [75, 76]. The SCO interlayer can be widely employed for the epitaxial growth of many functional complex oxide perovskites, e.g., STO, SRO, LSMO, BTO, and PbZr_*x*_Ti_1−*x*_O_3_ (PZT), BiFeO_3_ (BFO), and other binary oxides (e.g., CeO_2_, HfO_2_, and VO_2_) for fabricating high-quality freestanding complex oxide membranes. Another widely used complex oxide sacrificial layer is LSMO. By using the LSMO sacrificial layer, the epitaxial film growth of larger lattice-parameter perovskite dielectric and ferroelectric complex oxides, e.g., STO, PZT, and BFO, has been facilitated [77–79]. In the heterostructures, the LSMO layer can be selectively etched using various etchants, e.g., concentrated HF, ammonia-buffered HF acid (BHF), and a potassium iodide/hydrochloric acid (KI/HCl) solution in water [73, 80–82]. This can mainly be driven by reducing the insoluble Mn^4+^ to more soluble Mn^2+^ as described in the following reaction, MnO^2^ + 4H_3_O^+^ + 2Cl^−^ → Mn^2+^  + 6H_2_O + Cl_2_. However, the etching rate of the sacrificial LSMO layers becomes significantly slow, e.g., 12 h–24 h, once the etchants are highly diluted with water and aged [73, 83]. Recently, YBa_2_Cu_3_O_7−x_ (YBCO) has been proposed to be an alternative sacrificial layer as the copper oxide can be easily dissolved in HCl, denoted as CuO + 2HCl→Cu^2+^ + 2Cl^−^ + 2H_2_O [83]. The aggressive character of the chloride anion can boost the etching process of YBCO with a much faster etching rate (10 s for 120 nm-thick YBCO). Using the sacrificial YBCO layer, millimeter-scale freestanding LSMO and STO layers were successfully fabricated, exfoliating from STO substrates [83]. Additionally, zinc oxide (ZnO) is used as a sacrificial template layer that dissolves in HCl and supports the growth of mismatched substrates due to its epitaxial versatility [84, 85]. It has been recently shown that VO_2_ epitaxial films can be grown successfully on a ZnO layer on a wafer scale [85]. Moreover, other non-perovskite sacrificial layers have been explored, such as using MgO to release spinel CoFe_2_O_4_ membranes [86], followed by etching in H_3_SO_4_ solution. These developments highlight new methods for creating freestanding membranes from various oxide compositions through the chemical etching of epitaxial sacrificial layers. However, concentrated etchants (e.g., KI/KCl) can cause part of the damage to the desired complex oxide film materials [74, 83]. In addition, to mitigate the aggressiveness of the concentrated etchants, they often need to be diluted with DI water, and/or a DI water-rinsing process is subsequently required after the chemical etching to remove etchant residues. Therefore, it is crucial to carefully balance material selectivity, etching rates, and water dilution when employing chemical etchants to manufacture freestanding membranes of water-sensitive functional complex oxides. This is essential to maintain the functional properties of these materials.

## Conclusion and outlook

The future development of epitaxial film materials on Si-based substrates/device structures, particularly in the semiconductor and energy sectors, holds immense potential alongside recent advances in epitaxial lift-off techniques for freestanding functional complex oxide film membranes. However, for the advance of this field, several key challenges must be addressed and overcome:

1. **Material Compatibility and Sensitivity:** The primary challenges are the limited selection of materials imposed by a sacrificial material and sensitivity to water, air, and other solvents typically used in the membrane transfer process. Future research should focus on developing alternative sacrificial layers and/or water-free lift-off methods that do not compromise the functional properties of freestanding complex oxide film layers during film epitaxy and exfoliation processes, thereby expanding the range of usable materials.
2. **Improvement of Transfer Techniques:** While the current techniques have enabled the creation of ultrathin freestanding oxide films, there is a strong demand for further refinements to improve the yield, quality, and reproducibility of these films, e.g., millimeter-to-centimeter sizes and the absence of microcracks and wrinkles. Innovations in mechanical support and handling techniques, such as stamping polymers and thermal/chemical release on thin films, are required to reduce the incidence of undesired breakage, microcracks, and structural damage during the transfer process.
3. **Compatibility with Existing Technologies:** Integrating these advanced sacrificial materials into existing manufacturing processes remains challenging. Future developments should make these techniques compatible with standard semiconductor processes to facilitate broader adoption.
4. **Scalability**: There is an increasing interest in fabricating large-area freestanding complex oxide film layers from their epitaxial platforms for technological applications. Many of the current chemical lift-off techniques (e.g., using water-soluble sacrificial layers) are not yet scalable for industrial production. To the best of our knowledge, even millimeter-sized freestanding membranes have rarely been produced without microcracks and wrinkles. Research should aim to develop both epitaxial growth techniques, e.g., minimizing epitaxial strain with a sacrificial layer and large-area epitaxial film growth, and chemical lift-off methods, e.g., appropriately increasing dissolution rates and improving handling precision, for large-scale crack-free membrane fabrication without compromising the quality and functionality of the single-crystalline films [43]. In this context, remote epitaxy offers advantages for rapid mechanical lift-off and the production of larger-area oxide membranes, e.g., 5 × 5 mm^2^ [44]. To broaden the application of remote epitaxy across various complex oxides, development is needed to ensure additional growth methods, such as PLD, sputtering, and atomic layer deposition (ALD), since remote epitaxy is still highly sensitive to even minor imperfections such as the oxidation and surface modification of the layers. Therefore, techniques to transfer large-scale, high-quality, and uniform 2D monolayers/multilayers to various substrates must be developed.
5. **Control of Material Properties:** Precise control over the properties of the epitaxial films grown on these sacrificial layers is crucial. Most sacrificial layers significantly limit the subsequent film growth of upper oxide materials. Thus, it is vital to select an appropriate sacrificial layer or lift-off method that has a compatible growth window with the growth parameters of the main oxide films. This compatibility allows fine-tuning film functionalities by adjusting the growth parameters.
6. **Environmental and Economic Sustainability:** Future research should also consider the environmental impact of the new sacrificial materials and techniques, seeking to minimize waste and energy consumption. This includes environmentally friendly and non-toxic materials and chemicals and ensures easy and simple accessibility. Additionally, cost-effective methods need to be developed to ensure economic viability.

## Data Availability

The data that support the findings of this study are available from the corresponding author upon reasonable request.

## References

[1] G.E. Moore, Cramming more components onto integrated circuits. Electronics **38**, 8 (1965)

[2] S.E. Thompson, S. Parthasarathyk, Moore’s law: the future of Si microelectronics. Mater. Today **9**, 20–25 (2006)

[3] S. Veeraraghavan, J.G. Fossum, Short-channel effects in SOI MOSFETs. IEEE Trans. Electron Dev. **36**, 522–528 (1989)

[4] S.E. Thompson, R.S. Chau, T. Ghani, K. Mistry, S. Tyagi, M.T. Bohr, In search of “Forever,” continued transistor scaling one new material at a time. IEEE Trans. Semicond. Manufact. **18**, 26–36 (2005)

[5] E. Pop, S. Shinha, K.E. Goodson, Heat generation and transport in nanometer-scale transistors. Proc. IEEE **94**, 1587–1601 (2006)

[6] R.-H. Yan, A. Ourmazd, K.F. Lee, Scaling the Si MOSFET: from bulk to SOI to bulk. IEEE Trans. Electron Dev. **39**, 1704–1710 (1992)

[7] T. Khan, D. Vasileska, T.J. Thornton, Effect of interface roughness on silicon-on-insulator–metal-semiconductor field-effect transistor mobility and the device low-power high-frequency operation. J. Vac. Sci. Technol. B **23**, 1782–1784 (2005)

[8] M.U. Erdogan, M.C. Chang, C. Bowen, A. Chatterjee, J. Seitchik, H. Shichijo, Analysis of gate tunneling current in ultra-thin oxide MOSFET’s. 56th Annual Device Research Conference Digest (Cat. No.98TH8373), pp 14–15 (1998)

[9] S. Thompson, N. Anand, M. Armstrong, C. Auth, B. Arcot, M. Alavi, P. Bai, I. Bielefeld, R. Bigwood, J. Brandenburg, M. Buehler, S. Cea, V. Chikarmane, C. Choi, R. Frankovic, T. Ghani, G. Glass, W. Han, T. Hoffmann, M. Hussein, P. Jacob, A. lain. C. Jan, S. Joshi, C. Kenyon, I. Klaus, S Klopcic, I. Luce, Z. Ma, B. Mcintyre, K. Misty, A. Munhy, P. Nguyen. H. Pearson, T. Sandford, R. Schweinfunh, R. Shaheed, S. Sivakumar, M. Taylor, B. Tufts, C. Wallace, P. Wang, C. Weber, M. Bohr, A 90-nm logic technology featuring 50-nm strained silicon channel transistors, 7 layers of Cu interconnects, low k ILD, and1 µm^2^ SRAM cell. Tech. Dig. IEDM, pp 61–64 (2002)

[10] S.W. Bedell, A. Majumdar, J.A. Ott, J. Arnold, K. Fogel, S.J. Koester, D.K. Sadana, Mobility scaling in short-channel length strained Ge-on-Insulator P-MOSFETs. IEEE Electron Dev. Lett. **29**, 811–813 (2008)

[11] J. Robertson, R.M. Wallace, High-K materials and metal gates for CMOS applications. Mater. Sci. Eng. R **88**, 1–41 (2015)

[12] D. Hisamoto, W.-C. Lee, J. Kedzierski, H. Takeuchi, K. Asano, Ch. Kuo, E. Anderson, T.-J. King, J. Bokor, FinFET—a self-aligned double-gate MOSFET scalable to 20 nm. IEEE Trans. Electron Dev. **47**, 2320–2325 (2000)

[13] N. Singh, A. Agarwal, L.K. Bera, T.Y. Liow, R. Yang, S.C. Rustagi, C.H. Tung, R. Kumar, G.Q. Lo, N. Balasubramanian, D.-L. Kwong, High-performance fully depleted silicon nanowire (diameter ≤ 5 nm) gate-all-around CMOS devices. IEEE Electron Dev. Lett. **27**, 383–386 (2006)

[14] Reduced size, increased performance: Samsung’s GAA transistor, MBCFET™ (2019), https://news.samsung.com/global/infographic-reduced-size-increased-performancesamsungs-gaa-transistor-mbcfettm

[15] International Roadmap for Devices and Systems (IRDS^TM^) 2023 Edition (IEEE, 2023).

[16] F.M. Chiabrera, S. Yun, Y. Li, R.T. Dahm, H. Zhang, C.K.R. Kirchert, D.V. Christensen, F. Trier, T.S. Jespersen, N. Pryds, Freestanding perovskite oxide films: synthesis, challenges, and properties. Ann. Phys. **534**, 2200084 (2022)

[17] Z. Lu, Y. Yang, L. Wen, J. Feng, B. Lao, X. Zheng, S. Li, K. Zhao, B. Cao, Z. Ren, D. Song, H. Du, Y. Guo, Z. Zhong, X. Hao, Z. Wang, R.-W. Li, Cooperative control of perpendicular magnetic anisotropy via crystal structure and orientation in freestanding SrRuO_3_ membranes. Npj Flex. Electron. **6**, 9 (2022)

[18] J.-K. Huang, Y. Wan, J. Shi, J. Zhang, Z. Wang, W. Wang, N. Yang, Y. Liu, C.-H. Lin, X. Guan, L. Hu, Z.-L. Yang, B.-C. Huang, Y.-P. Chiu, J. Yang, V. Tung, D. Wang, K. Kalantar-Zadeh, T. Wu, X. Zu, L. Qiao, L.-J. Li, S. Li, High-*κ* perovskite membranes as insulators for two-dimensional transistors. Nature **605**, 262–267 (2022) 35546188 10.1038/s41586-022-04588-2

[19] C. Lu, M. Li, L. Gao, Q. Zhang, M. Zhu, X. Lyu, Y. Wang, J. Liu, P. Liu, L. Wang, H. Tao, J. Song, A. Ji, P. Li, L. Gu, Z. Cao, N. Lu, Freestanding crystalline *β*-Ga_2_O_3_ flexible membrane obtained via lattice epitaxy engineering for high-performance optoelectronic device. ACS Nano **18**, 5374–5382 (2024) 10.1021/acsnano.3c10025 38335925

[20] P. Su, H. Wen, Y. Zhang, C. Tan, X. Zhong, Y. Wu, H. Song, Y. Zhou, Y. Li, M. Liu, J. Wang, Super-flexibility in freestanding single-crystal SrRuO_3_ conductive oxide membranes. ACS Appl. Electron. Mater. **4**, 2987–2992 (2022)

[21] B. Peng, R.-C. Peng, Y.-Q. Zhang, G. Dong, Z. Zhou, Y. Zhou, T. Li, Z. Liu, Z. Luo, S. Wang, Y. Xia, R. Qiu, X. Cheng, F. Xue, Z. Hu, W. Ren, Z.-G. Ye, L.-Q. Chen, Z. Shan, T. Min, M. Liu, Phase transition enhanced superior elasticity in freestanding single-crystalline multiferroicBiFeO_3_ membranes. Sci. Adv. **6**, eaba5847 (2020) 32937363 10.1126/sciadv.aba5847 PMC7442355

[22] Y. Guo, B. Peng, G. Lu, G. Dong, G. Yang, B. Chen, R. Qiu, H. Liu, B. Zhang, Y. Yao, Y. Zhao, S. Li, X. Ding, J. Sun, M. Liu, Remarkable flexibility in freestanding single-crystalline antiferroelectric PbZrO_3_ membranes. Nat. Commun. **15**, 4414 (2024) 38782889 10.1038/s41467-024-47419-w PMC11116490

[23] Y. Shen, K. Ooe, X. Yuan, T. Yamada, S. Kobayashi, M. Haruta, D. Kan, Y. Shimakawa, Ferroelectric freestanding hafnia membranes with metastable rhombohedral structure down to 1-nm-thick. Nat. Commun. **15**, 4789 (2024) 38918364 10.1038/s41467-024-49055-w PMC11199652

[24] J. Jiang, L. Zhang, C. Ming, H. Zhou, P. Bose, Y. Guo, Y. Hu, B. Wang, Z. Chen, R. Jia, S. Pendse, Y. Xiang, Y. Xia, Z. Lu, X. Wen, Y. Cai, C. Sun, G.-C. Wang, T.-M. Lu, D. Gall, Y.-Y. Sun, N. Koratkar, E. Fohtung, Y. Shi, J. Shi, Giant pyroelectricity in nanomembranes. Nature **607**, 480–485 (2022) 35859196 10.1038/s41586-022-04850-7

[25] G. Sánchez-Santolino, V. Rouco, S. Puebla, H. Aramberri, V. Zamora, M. Cabero, F.A. Cuellar, C. Munuera, F. Mompean, M. Garcia-Hernandez, A. Castellanos-Gomez, J. Íñiguez, C. Leon, J. Santamaria, A 2D ferroelectric vortex pattern in twisted BaTiO_3_ freestanding layers. Nature **626**, 529–534 (2024) 38356067 10.1038/s41586-023-06978-6 PMC10866709

[26] N. Pyds, D.-S. Park, T.S. Jespersen, S. Yun, Twisted oxide membranes: a perspective. APL Mater. **12**, 010901 (2024)

[27] S.H. Baek, J. Park, D.M. Kim, V.A. Aksyuk, R.R. Das, S.D. Bu, D.A. Felker, J. Lettieri, V. Vaithyanathan, S.S.N. Bharadwaja, N. Bassiri-Gharb, Y.B. Chen, H.P. Sun, C.M. Folkman, H.W. Jang, D.J. Kreft, S.K. Streiffer, R. Ramesh, X.Q. Pan, S. Trolier-McKinstry, D.G. Schlom, M.S. Rzchowski, R.H. Blick, C.B. Eom, Giant piezoelectricity on Si for hyperactive MEMS. Science **334**, 958 (2011) 22096193 10.1126/science.1207186

[28] J. Li, J. Wang, M. Wuttig, R. Ramesh, N. Wang, B. Ruette, A.P. Pyatakov, A.K. Zvezdin, D. Viehland, Dramatically enhanced polarization in (001), (101), and (111) BiFeO_3_ thin films due to epitiaxial-induced transitions. Appl. Phys. Lett. **84**, 5261–5263 (2004)

[29] I. Vrejoiu, G. Le Rhun, L. Pintilie, D. Hesse, M. Alexe, U. Gösele, Intrinsic ferroelectric properties of strained tetragonal PbZr_0.2_Ti_0.8_O_3_ obtained on layer–by–layer grown, defect-free single–crystalline films. Adv. Mater. **18**, 1657–1661 (2006)

[30] A. Schilling, M. Cantoni, J.D. Guo, H.R. Ott, Superconductivity above 130 K in the Hg–Ba–Ca–Cu–O system. Nature **363**, 56–58 (1993)

[31] R. Gao, A.C.P. Jain, S. Pandya, Y. Dong, Y. Yuan, H. Zhou, L.R. Dedon, V. Thoréton, S. Saremi, R. Xu, A. Luo, T. Chen, V. Gopalan, E. Ertekin, J. Kilner, T. Ishihara, N.H. Perry, D.R. Trinkle, L.W. Martin, Designing optimal perovskite structure for high ionic conduction. Adv. Mater. **32**, 1905178 (2020) 10.1002/adma.201905178 31680355

[32] C. Huo, K. Xu, L. Ma, T. Li, H. Li, X. Yang, X. Kuang, S. Liu, S. Deng, J. Chen, Colossal ionic conductivity in interphase strain-engineered nanocomposite films. J. Am. Chem. Soc. **145**, 13623–13631 (2023) 37327186 10.1021/jacs.3c01298

[33] S. Jin, M. McCormack, T.H. Tiefel, R. Ramesh, Colossal magnetoresistance in La-Ca-Mn-O ferromagnetic thin films. J. Appl. Phys. **76**, 6929–6933 (1994)

[34] D.-S. Park, A.D. Rata, I.V. Maznichenko, S. Ostanin, Y.L. Gan, S. Agrestini, G.J. Rees, M. Walker, J. Li, J. Herrero-Martin, G. Singh, Z. Luo, A. Bhatnagar, Y.Z. Chen, V. Tileli, P. Muralt, A. Kalaboukhov, I. Mertig, K. Dörr, A. Ernst, N. Pryds, The emergence of magnetic ordering at complex oxide interfaces tuned by defects. Nat. Commun. **11**, 3650 (2020) 32686663 10.1038/s41467-020-17377-0 PMC7371687

[35] A.D. Rata, J. Herrero-Martin, I.V. Maznichenko, F.M. Chiabrera, R.T. Dahm, S. Ostanin, D. Lee, B. Jalan, P. Buczek, I. Mertig, A. Ernst, A.M. Ionescu, K. Dörr, N. Pryds, D.-S. Park, Defect-induced magnetism in homoepitaxial SrTiO_3_. APL Mater. **10**, 091108 (2022)

[36] J. Jeong, N. Aetukuri, T. Graf, T.D. Schladt, M.G. Samant, S.S.P. Parkin, Suppression of metal-insulator transitionin VO_2_ by electric field–induced oxygen vacancy formation. Science **339**, 1402–1405 (2013) 23520104 10.1126/science.1230512

[37] J. Wang, J.B. Neaton, H. Zheng, V. Nagarajan, S.B. Ogale, B. Liu, D. Viehland, V. Vaithyanathan, D.G. Schlom, U.V. Waghmare, N.A. Spaldin, K.M. Rabe, M. Wuttig, R. Ramesh, Epitaxial BiFeO_3_ multiferroic thin film heterostructures. Science **299**, 1719–1722 (2003) 12637741 10.1126/science.1080615

[38] D. Lu, D.J. Baek, S.S. Hong, L.F. Kourkoutis, Y. Hikita, H.Y. Hwang, Synthesis of freestanding single-crystal perovskite films and heterostructures by etching of sacrificial water-soluble layers. Nat. Mater. **15**, 1255–1260 (2016) 27618712 10.1038/nmat4749

[39] Q. Wang, H. Liu, B. He, J. Qi, D. Wang, H. Xu, N. Zhang, J. Wang, Y. Chen, Z. Wang, Z. Wang, X. Qi, L. Zheng, M. Feng, W. Lü, S. Yan, Enhanced oxygen evolution reaction by stacking single-crystalline freestanding SrRuO_3_. Appl. Catal. B **317**, 121781 (2022)

[40] D. Kim, W.K. Jung, S. Lee, Single-crystalline-level properties of ultrathin SrRuO_3_ flexible membranes with wide and clean surface. Npj Flex. Electron. **6**, 24 (2022)

[41] P. Salles, R. Guzman, A. Barrera, M. Ramis, J.M. Caicedo, A. Palau, W. Zhou, M. Coll, On the role of the Sr_3−x_Ca_x_Al_2_O_6_ sacrificial layer composition in epitaxial La_0.7_Sr_0.3_MnO_3_ membranes. Adv. Funct. Mater. **33**, 2304059 (2023)

[42] S. Yun, T.E. Cozannet, C.H. Christoffersen, E. Brand, T.S. Jespersen, N. Pryds, Strain engineering: perfecting freestanding perovskite oxide fabrication. Small **20**, 2310782 (2024) 10.1002/smll.202310782 38431927

[43] J. Zhang, T. Lin, A. Wang, X. Wang, Q. He, H. Ye, J. Lu, Q. Wang, Z. Liang, F. Jin, S. Chen, M. Fan, E.-J. Guo, Q. Zhang, L. Gu, Z. Luo, L. Si, W. Wu, L. Wang, Super-tetragonal Sr_4_Al_2_O_7_ as a sacrificial layer for high-integrity freestanding oxide membranes. Science **383**, 388–394 (2024) 38271502 10.1126/science.adi6620

[44] H.S. Kum, H. Lee, S. Kim, S. Lindemann, W. Kong, K. Qiao, P. Chen, J. Irwin, J.H. Lee, S. Xie, S. Subramanian, J. Shim, S.-H. Bae, C. Choi, L. Ranno, S. Seo, S. Lee, J. Bauer, H. Li, K. Lee, J.A. Robinson, C.A. Ross, D.G. Schlom, M.S. Rzchowski, C.-B. Eom, J. Kim, Heterogeneous integration of single-crystalline complex-oxide membranes. Nature **578**, 75–81 (2020) 32025010 10.1038/s41586-020-1939-z

[45] J. Kim, C. Bayram, H. Park, C.-W. Cheng, C. Dimitrakopoulos, J.A. Ott, K.B. Reuter, S.W. Bedell, D.K. Sadana, Principle of direct van der Waals epitaxy of single-crystalline films on epitaxial graphene. Nat. Commun. **5**, 4836 (2014) 25208642 10.1038/ncomms5836

[46] K. Qiao, Y. Liu, C. Kim, R.J. Molnar, T. Osadchy, W. Li, X. Sun, H. Li, R.L. Myers-Ward, D. Lee, S. Subramanian, H. Kim, K. Lu, J.A. Robinson, W. Kong, J. Kim, Graphene buffer layer on SiC as a release layer for high-quality freestanding semiconductor membranes. Nano Lett. **21**, 4013–4020 (2021) 33900785 10.1021/acs.nanolett.1c00673

[47] Y. Tchoe, K. Chung, K. Lee, J. Jo, K. Chung, J.K. Hyun, M. Kim, G.-C. Yi, Free-standing and ultrathin inorganic light-emitting diode array. NPG Asia Mater. **11**, 37 (2019)

[48] F. Liu, T. Wang, X. Gao, H. Yang, Z. Zhang, Y. Guo, Y. Yuan, Z. Huang, J. Tang, B. Sheng, Z. Chen, K. Liu, B. Shen, X.-Z. Li, H. Peng, X. Wang, Determination of the preferred epitaxy for III-nitride semiconductors on wet-transferred graphene. Sci. Adv. **9**, eadf8484 (2023) 37531436 10.1126/sciadv.adf8484 PMC10396303

[49] Y. Kim, S.S. Cruz, K. Lee, B.O. Alawode, C. Choi, Y. Song, J.M. Johnson, C. Heidelberger, W. Kong, S. Choi, K. Qiao, I. Almansouri, E.A. Fitzgerald, J. Kong, A.M. Kolpak, J. Hwang, J. Kim, Remote epitaxy through graphene enables two-dimensional material-based layer transfer. Nature **544**, 340–343 (2017) 28426001 10.1038/nature22053

[50] H. Kim, S. Lee, J. Shin, M. Zhu, M. Akl, K. Lu, N.M. Han, Y. Baek, C.S. Chang, J.M. Suh, K.S. Kim, B.-I. Park, Y. Zhang, C. Choi, H. Shin, H. Yu, Y. Meng, S.-I. Kim, S. Seo, K. Lee, H.S. Kum, J.-H. Lee, J.-H. Ahn, S.-H. Bae, J. Hwang, Y. Shi, J. Kim, Graphene nanopattern as a universal epitaxy platform for single-crystal membrane production and defect reduction. Nat. Nanotechnol. **17**, 1054–1059 (2022) 36138198 10.1038/s41565-022-01200-6

[51] K.M. Adkison, S.-L. Shang, B.J. Bocklund, D. Klimm, D.G. Schlom, Z.-K. Liu, Suitability of binary oxides for molecular-beam epitaxy source materials: a comprehensive thermodynamic analysis. APL Mater. **8**, 081110 (2020)

[52] H. Yoon, T.K. Truttmann, F. Liu, B.E. Matthews, S. Choo, Q. Su, V. Saraswat, S. Manzo, M.S. Arnold, M.E. Bowden, J.K. Kawasaki, S.J. Koester, S.R. Spurgeon, S.A. Chambers, B. Jalan, Freestanding epitaxial SrTiO_3_ nanomembranes via remote epitaxy using hybrid molecular beam epitaxy. Sci. Adv. **8**, eadd5328 (2022) 36563139 10.1126/sciadv.add5328 PMC9788776

[53] M.A. Wohlgemuth, U. Trstenjak, A. Sarantopoulos, F. Gunkel, R. Dittmann, Control of growth kinetics during remote epitaxy of complex oxides on graphene by pulsed laser deposition. APL Mater. **12**, 021113 (2024)

[54] D.K. Lee, Y. Park, H. Sim, J. Park, Y. Kim, G.-Y. Kim, C.-B. Eom, S.-Y. Choi, J. Son, Heterogeneous integration of single-crystalline rutile nanomembranes with steep phase transition on silicon substrates. Nat. Commun. **12**, 5019 (2021) 34408136 10.1038/s41467-021-24740-2 PMC8373986

[55] D.-S. Park, M. Hadad, L.M. Riemer, R. Ignatans, D. Spirito, V. Esposito, V. Tileli, N. Gauquelin, D. Chezganov, D. Jannis, J. Verbeeck, S. Gorfman, N. Pryds, P. Muralt, D. Damjanovic, Induced giant piezoelectricity in centrosymmetric oxides. Science **375**, 653–657 (2022) 35143321 10.1126/science.abm7497

[56] M.-J. Lee, C.B. Lee, D. Lee, S.R. Lee, M. Chang, J.H. Hur, Y.-B. Kim, C.-J. Kim, D.H. Seo, S. Seo, U. Chung, I.-K. Yoo, K. Kim, A fast, high-endurance and scalable non-volatile memory device made from asymmetric Ta_2_O_5−__*x*_/TaO_2−__*x*_ bilayer structures. Nat. Mater. **10**, 625–630 (2011) 21743450 10.1038/nmat3070

[57] F. Bi, D.F. Bogorin, C. Cen, C.W. Bark, J.-W. Park, C.-B. Eom, J. Levy, “Water-cycle” mechanism for writing and erasing nanostructures at the LaAlO_3_/SrTiO_3_ interface. Appl. Phys. Lett. **97**, 173110 (2010)

[58] H. Lee, T.H. Kim, J.J. Patzner, H. Lu, J.-W. Lee, H. Zhou, W. Chang, M.K. Mahanthappa, E.Y. Tsymbal, A. Gruverman, C.-B. Eom, Imprint control of BaTiO_3_ thin films via chemically induced surface polarization pinning. Nano Lett. **16**, 2400–2406 (2016) 26901570 10.1021/acs.nanolett.5b05188

[59] Y. Tian, L. Wei, Q. Zhang, H. Huang, Y. Zhang, H. Zhou, F. Ma, L. Gu, S. Meng, L.-Q. Chen, C.-W. Nan, J. Zhang, Water printing of ferroelectric polarization. Nat. Commun. **9**, 3809 (2018) 30228308 10.1038/s41467-018-06369-w PMC6143547

[60] T.-A. Chen, C.-P. Chuu, C.-C. Tseng, C.-K. Wen, H.-S.P. Wong, S. Pan, R. Li, T.-A. Chao, W.-C. Chueh, Y. Zhang, Q. Fu, B.I. Yakobson, W.-H. Chang, L.-J. Li, Wafer-scale single-crystal hexagonal boron nitride monolayers on Cu (111). Nature **579**, 219–223 (2020) 32132712 10.1038/s41586-020-2009-2

[61] M. Mojtabavi, A. VahidMohammadi, K. Ganeshan, D. Hejazi, S. Shahbazmohamadi, S. Kar, A.C.T. van Duin, M. Wanunu, Wafer-scale lateral self-assembly of mosaic Ti_3_C_2_T_*x*_ MXene monolayer films. ACS Nano **15**, 625–636 (2021) 33405898 10.1021/acsnano.0c06393

[62] D.P. Kumah, J.H. Ngai, L. Kornblum, Epitaxial oxides on semiconductors: from fundamentalsto new devices. Adv. Funct. Mater. **30**, 1901597 (2020)

[63] https://www.universitywafer.com/silicon-wafer-price.html?srsltid=AfmBOoovqMCxFkoruXbNP6362_Q4FnrVKtNgHSDsN0zIMPuljsMPWOWQ

[64] S.-H. Baek, C.-B. Eom, Epitaxial integration of perovskite-based multifunctional oxides on silicon. Acta Mater. **61**, 2734–2750 (2013)

[65] J.W. Park, D.F. Bogorin, C. Cen, D.A. Felker, Y. Zhang, C.T. Nelson, C.W. Bark, C.M. Folkman, X.Q. Pan, M.S. Rzchowski, J. Levy, C.B. Eom, Creation of a two-dimensional electron gas at an oxide interface on silicon. Nat. Commun. **1**, 94 (2010) 20981022 10.1038/ncomms1096

[66] G. Niu, B. Vilquin, J. Penuelas, C. Botella, G. Hollinger, G. Saint-Girons, Heteroepitaxy of SrTiO_3_ thin films on Si (001) using different growth strategies: toward substratelike quality. J. Vac. Sci. Technol. B **29**, 041207 (2011)

[67] Z. Jovanović, U. Trstenjak, H.-C. Ho, O. Butsyk, B. Chen, E. Tchernychova, F. Borodavka, G. Koster, J. Hlinka, M. Spreitzer, Tiling the silicon for added functionality: PLD growth of highly crystalline STO and PZT on graphene oxide-buffered silicon surface. ACS Appl. Mater. Interfaces **15**, 6058–6068 (2023) 36653314 10.1021/acsami.2c17351 PMC9906728

[68] H. Li, S. Yun, A. Chikina, V. Rosendal, T. Tran, E. Brand, C.H. Christoffersen, N.C. Plumb, M. Shi, N. Pryds, M. Radovic, Transition metal-oxide nanomembranes assembly by direct Heteroepitaxial growth. Adv. Funct. Mater. **34**, 2313236 (2024)

[69] J. Anguita, F. Briones, HF/H_2_O vapor etching of SiO_2_ sacrificial layer for large-area surface-micromachined membranes. Sens. Actuator A **64**, 247–251 (1998)

[70] E. Yablonovitch, T. Gmltter, J.P. Harbison, R. Bhat, Extreme selectivity in the lift-off of epitaxial GaAs films. Appl. Phys. Lett. **51**, 2222–2224 (1987)

[71] C.-W. Cheng, K.-T. Shiu, N. Li, S.-J. Han, L. Shi, D.K. Sadana, Epitaxial lift-off process for gallium arsenide substrate reuse and flexible electronics. Nat. Commun. **4**, 1577 (2013) 23481385 10.1038/ncomms2583

[72] Q. Gan, R.A. Rao, C.B. Eom, J.L. Garrett, M. Lee, Direct measurement of strain effects on magnetic and electrical properties of epitaxial SrRuO_3_ thin films. Appl. Phys. Lett. **72**, 978–980 (1998)

[73] D. Weber, R. Vőfély, Y. Chen, Y. Mourzina, U. Poppe, Variable resistor made by repeated steps of epitaxial deposition and lithographic structuring of oxide layers by using wet chemical etchants. Thin Solid Films **533**, 43–47 (2013)

[74] D. Pesquera, E. Khestanova, M. Ghidini, S. Zhang, A.P. Rooney, F. Maccherozzi, P. Riego, S. Farokhipoor, J. Kim, X. Moya, M.E. Vickers, N.A. Stelmashenko, S.J. Haigh, S.S. Dhesi, N.D. Mathur, Large magnetoelectric coupling in multiferroic oxide heterostructures assembled via epitaxial lift-off. Nat. Commun. **11**, 3190 (2020) 32581280 10.1038/s41467-020-16942-x PMC7314756

[75] H. Peng, N. Lu, S. Yang, Y. Lyu, Z. Liu, Y. Bu, S. Shen, M. Li, Z. Li, L. Gao, S. Lu, M. Wang, H. Cao, H. Zhou, P. Gao, H. Chen, P. Yu, A generic sacrificial layer for wide-range freestanding oxides with modulated magnetic anisotropy. Adv. Funct. Mater. **32**, 2111907 (2022)

[76] W. Zhou, W. Han, Y. Yang, L. Shu, Q. Luo, Y. Ji, C. Jin, Y. Zhang, J. Song, M. Ye, Q. Liu, S. Hu, L. Chen, Synthesis of freestanding perovskite oxide thin films by using brownmillerite SrCoO_2.5_ as a sacrificial layer. Appl. Phys. Lett. **122**, 062901 (2023)

[77] Y. Zhang, J. Li, K. Yang, F. Zheng, Y. Zhou, Y. Zhang, Y. Hui, Y.-Q. Wang, J. Zhu, J. Zhang, Y. Hao, M. Yang, T. Li, J. Zhao, H.H. Ma, Room-temperature electric field-induced out-of-plane ferroelectric polarization in strain-free freestanding 2D SrTiO_3_ membranes. APL Mater. **11**, 041103 (2023)

[78] S.R. Bakaul, C.R. Serrao, M. Lee, C.W. Yeung, A. Sarker, S.-L. Hsu, A.K. Yadav, L. Dedon, L. You, A.I. Khan, J.D. Clarkson, C. Hu, R. Ramesh, S. Salahuddin, Single crystal functional oxides on silicon. Nat. Commun. **7**, 10547 (2016) 26853112 10.1038/ncomms10547 PMC4748113

[79] P.-C. Wu, C.-C. Wei, Q. Zhong, S.-Z. Ho, Y.-D. Liou, Y.-C. Liu, C.-C. Chiu, W.-Y. Tzeng, K.-E. Chang, Y.-W. Chang, J. Zheng, C.-F. Chang, C.-M. Tu, T.-M. Chen, C.-W. Luo, R. Huang, C.-G. Duan, Y.-C. Chen, C.-Y. Kuo, J.-C. Yang, Twisted oxide lateral homostructures with conjunction tunability. Nat. Commun. **13**, 2565 (2022) 35538081 10.1038/s41467-022-30321-8 PMC9090740

[80] R.M. Stroud, J. Kim, C.R. Eddy, D.B. Chrisey, J.S. Horwitz, D. Koller, M.S. Osofsky, R.J. Soulen Jr., R.C.Y. Auyeung, Fabrication of YBa_2_Cu_3_O_7-δ_/SrTiO_3_/La_0.7_Sr_0.3_MnO_3-δ_ junctions for the control of supercurrent by spin-polarized quasiparticle current injection. J. Appl. Phys. **83**, 7189–7191 (1998)

[81] S.-S. Lee, D.-G. Hwang, K. Rhie, Magnetoresistive La_0.8_Sr_0.2_MnO_3-δ_ biepitaxial films grown on Al_2_O_3_ (1 1 20) substrates. J. Korean Phys. Soc. **37**, 283–286 (2000)

[82] J.-H. Kim, A.M. Grishin, V.A. Ignatova, Wet etching study of La_0.67_(Sr_0.5_Ca_0.5_)_0.33_MnO3 films on silicon substrates. J. Electron. Mater. **37**, 361–367 (2008)

[83] Y.-W. Chang, P.-C. Wu, J.-B. Yi, Y.-C. Liu, Y. Chou, Y.-C. Chou, J.-C. Yang, A fast route towards freestanding single-crystalline oxide thin films by using YBa_2_Cu_3_O_7-x_ as a sacrificial layer. Nanoscale Res. Lett. **15**, 172 (2020) 32857192 10.1186/s11671-020-03402-0 PMC7455685

[84] D.J. Rogers, F. Hosseini Teherani, A. Ougazzaden, S. Gautier, L. Divay, A. Lusson, O. Durand, F. Wyczisk, G. Garry, T. Monteiro, M.R. Correira, M. Peres, A. Neves, D. McGrouther, J.N. Chapman, M. Razeghi, Use of ZnO thin films as sacrificial templates for metal organic vapor phase epitaxy and chemical lift-off of GaN. Appl. Phys. Lett. **91**, 071120 (2007)

[85] X. Li, Z. Yin, X. Zhang, Y. Wang, D. Wang, M. Gao, J. Meng, J. Wu, J. You, Epitaxial liftoff of wafer-scale VO_2_ nanomembranes for flexible, ultrasensitive tactile sensors. Adv. Mater. Technol. **4**, 1800695 (2019)

[86] Y. Zhang, L. Shen, M. Liu, X. Li, X. Lu, L. Lu, C. Ma, C. You, A. Chen, C. Huang, L. Chen, M. Alexe, C.-L. Jia, Flexible quasi two dimensional CoFe_2_O_4_ epitaxial thin films for continuous strain tuning of magnetic properties. ACS Nano **11**, 8002–8009 (2017) 28657728 10.1021/acsnano.7b02637

